# A Systematic Review of Methods to Predict Weight Trajectories in Health Economic Models of Behavioral Weight-Management Programs: The Potential Role of Psychosocial Factors

**DOI:** 10.1177/0272989X19889897

**Published:** 2019-12-02

**Authors:** Sarah Bates, Thomas Bayley, Paul Norman, Penny Breeze, Alan Brennan

**Affiliations:** School of Health and Related Research, University of Sheffield, Sheffield, South Yorkshire, UK; School of Health and Related Research, University of Sheffield, Sheffield, South Yorkshire, UK; Department of Psychology, University of Sheffield, Sheffield, South Yorkshire, UK; School of Health and Related Research, University of Sheffield, Sheffield, South Yorkshire, UK; School of Health and Related Research, University of Sheffield, Sheffield, South Yorkshire, UK

**Keywords:** behavioral weight-management, cost-effectiveness analysis, health economic modeling, weight loss maintenance

## Abstract

**Objectives.** There is limited evidence on the long-term effectiveness of behavioral weight-management interventions, and thus, when conducting health economic modeling, assumptions are made about weight trajectories. The aims of this review were to examine these assumptions made about weight trajectories, the evidence sources used to justify them, and the impact of assumptions on estimated cost-effectiveness. Given the evidence that some psychosocial variables are associated with weight-loss trajectories, we also aimed to examine the extent to which psychosocial variables have been used to estimate weight trajectories and whether psychosocial variables were measured within cited evidence sources. **Methods.** A search of databases (Medline, PubMed, Cochrane, NHS Economic Evaluation, Embase, PSYCinfo, CINAHL, EconLit) was conducted using keywords related to overweight, weight-management, and economic evaluation. Economic evaluations of weight-management interventions that included modeling beyond trial data were included. **Results.** Within the 38 eligible articles, 6 types of assumptions were reported (weight loss maintained, weight loss regained immediately, linear weight regain, subgroup-specific trajectories, exponential decay of effect, maintenance followed by regain). Fifteen articles cited at least 1 evidence source to support the assumption reported. The assumption used affected the assessment of cost-effectiveness in 9 of the 19 studies that tested this in sensitivity analyses. None of the articles reported using psychosocial factors to estimate weight trajectories. However, psychosocial factors were measured in evidence sources cited by 11 health economic models. **Conclusions**. Given the range of weight trajectories reported and the potential impact on funding decisions, further research is warranted to investigate how psychosocial variables measured in trials can be used within health economic models to simulate heterogeneous weight trajectories and potentially improve the accuracy of cost-effectiveness estimates.

Behavioral weight-management programs are the first line of treatment recommended by the National Institute for Health and Care Excellence (NICE) for individuals who have a body mass index (BMI) of greater than 25 in England.^1^ Systematic evidence reviews and large clinical trials show that many of these programs are associated with significant weight loss,^2,3^ but the long-term success, as measured by lasting weight loss maintenance, is harder to determine. Although there are weight-management studies with a follow-up of up to 10 years or longer,^4,5^ most have a maximum of only 2 to 3 years.^6^ Moreover, the limited evidence available is mixed; while recent reviews have indicated that weight is regained by approximately 5 years,^6,7^ in an observational study based in the United States, participants (*n* > 4000) reported maintaining an average weight loss of 33 kg, from an original weight of 105 kg, for about 5.7 years.^8,9^

The lack of long-term data introduces additional uncertainty to decisions of whether to fund an intervention. One aspect considered in this decision making is cost-effectiveness analysis (CEA). Within CEA, health economic models (HEMs) can be used to extrapolate costs and effectiveness of weight-management programs beyond trial data to determine cost-effectiveness over a longer period of time.^10^ To conduct this analysis, an estimation of intervention effect is modeled,^11^ and, in the absence of long-term data, an assumption is made about weight trajectories beyond the trial period both with and without the intervention. For example, in the economic modeling conducted to inform NICE obesity guidelines, it was assumed that individuals regained 5% of the weight loss annually, resulting in a return to the nonintervention weight trajectory after 20 years.^12^ The assumption used is partly determined by the HEM structure used,^13^ which can allow for estimating either a mean weight trajectory for all individuals, weight trajectories for certain subgroups, or a weight trajectory for each individual. The assumption used determines the duration of benefits gained from an intervention, which will affect costs and consequences, the assessment of cost-effectiveness, and potentially the funding decision made.

Weight trajectories during and after weight-management interventions are likely to be affected by a variety of individual factors, and consideration of these factors could potentially improve the accuracy of assumptions made with HEMs and of resulting cost-effectiveness estimates. Psychosocial variables are considered to be important factors in obesity and are often the target for behavioral interventions.^14,15^ There is growing evidence of associations between psychosocial factors, such as self-regulation, motivation, self-efficacy and habit, and weight loss maintenance.^16–18^ In a review of experimental studies, higher internal motivation compared with motivation driven by external pressure, self-efficacy (an individual’s belief in their ability to change and maintain healthy behaviors), and self-regulation skills (e.g. monitoring of diet, exercise, weight and employing goal setting strategies) were predictive of weight loss.^17^ A positive body image, flexible dietary restraint (restriction of dietary intake),^16,17^ and habit (the extent to which healthy behaviors have become automatic) have also been associated with weight loss maintenance.^16^ Given there is strong evidence to indicate that psychosocial factors are important in weight trajectories, including these variables in HEMs has 2 potential benefits. First, in the absence of long-term data, these variables could be used to predict weight trajectories postintervention and represent the heterogeneity in weight trajectories. This has the potential to increase the accuracy of estimates of long-term cost-effectiveness. Second, HEMs could be used to estimate the impact of planned behavioral interventions that are expected to change certain psychosocial factors (e.g., a habit-based intervention^19^), and this can be used in the intervention design process.

There has been a broad review of HEMs used to estimate the cost-effectiveness of obesity prevention and treatment interventions,^20^ but none through September 2019 have specifically examined the assumptions made regarding weight trajectories. Given the potential impact of these assumptions on estimates of cost-effectiveness, the aims of this review are to examine 1) the assumptions that are made about weight trajectories within HEMs of behavioral weight-management interventions for overweight and obesity; 2) what, if any, evidence sources are used to justify these assumptions; and 3) the impact of differing assumptions on conclusions about cost-effectiveness. Furthermore, given that there is evidence to indicate that inclusion of psychosocial factors may contribute to accurate predictions of weight trajectories, this review will also document 4) which, if any, variables have been used to predict weight trajectories within HEMs and 5) whether psychosocial variables were measured within the evidence sources that informed the modeled weight trajectory.

## Method

PRISMA guidelines were followed when conducting this systematic review.^21^

### Study Searches

Searches were conducted in November 2017 in Medline, PubMed, Cochrane, National Health Service (NHS) economic evaluation (EE) database, Embase, PSYCinfo, CINAHL, and EconLit including terms related to overweight or obesity, weight loss management, and recommended search terms for economic evaluations^22^ with no restriction on year of publication (the full search strategy in available in Supplementary Appendix 2). The reference lists of eligible articles were searched and retrieved, and citation searches were conducted. The search was updated in July 2019 using the same search strategy to identify any recent studies published.

### Study Selection

Titles and abstracts were reviewed, and the full text of remaining articles was then screened to determine eligibility. A random selection (10%) of the full articles reviewed was screened by a second reviewer (T.B.), and any disagreements about eligibility were discussed. Studies were included if they reported an original economic evaluation (i.e., not a review of health economic evaluations or models) of at least 1 behavioral weight-management intervention aimed at adults (aged 18–65 years) who were above a healthy weight (i.e., with a BMI >25) with the aim of reducing weight. Studies also had to include modeling of weight trajectories beyond data available from the intervention trial. Studies were excluded if the intervention was aimed at a population with a health condition (this included diabetes, cancer, pregnancy, a history of recent surgery including bariatric surgery, and in rehabilitation from a recent cardiovascular event) that could have affected the weight trajectory or if more than half of the study sample had 1 of these conditions. The weight trajectories and the factors that affect these may differ for those with and without health conditions; for example, those with diabetes regain weight more quickly than those without.^6^ Studies were excluded if they did not include an evaluation of at least 1 behavioral weight-management intervention or if the behavioral weight-management intervention included a pharmacological or surgical component (e.g., weight-management intervention paired with a weight loss medication). Studies were excluded if they did not report a full economic evaluation; that is, if they did not include an assessment of both costs and outcomes and/or did not include a comparison of 2 or more interventions.^10^ Publications in languages other than English were excluded.

### Study Characteristics

A data extraction form (Supplementary Appendix 3) was used to extract details of the weight trajectory modeling methods. The assumptions made about weight trajectories, any cited evidence sources, and any sensitivity analysis conducted regarding the weight trajectory (and the related impact on outcomes) were extracted. Any psychosocial factors that had been used in the prediction of weight trajectories and the measurement and analysis of these factors within the articles and in cited evidence sources were also extracted.

### Data Synthesis

As this is a review of methods rather than an estimation of treatment effects, we did not undertake a meta-analysis of studies or assess studies for quality. A detailed review of methods and a narrative synthesis were conducted; assumptions made about weight trajectories within HEMs were categorized, and the evidence sources were examined and summarized. Any sensitivity analyses around the weight trajectory assumptions were reviewed and their effects on the incremental cost-effectiveness ratio (ICER) described. The psychosocial variables used within the HEMs or measured within evidence sources cited and any analysis conducted on these variables were summarized.

## Results

**Figure 1:**
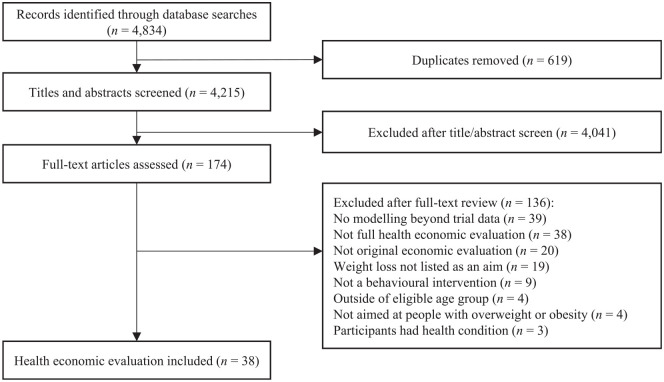
PRISMA flow diagram.

Including the original and updated search, 4215 titles and abstracts were reviewed. Of these, the full text of 174 articles were reviewed and 136 were excluded; the most common reasons were that the articles were not a full health economic evaluation or that there was no modeling beyond the trial data. A total of 38 studies (Supplementary Appendix 1) met the eligibility criteria (Figure 1).

### Assumptions Made about Weight Trajectories

**Figure 2:**
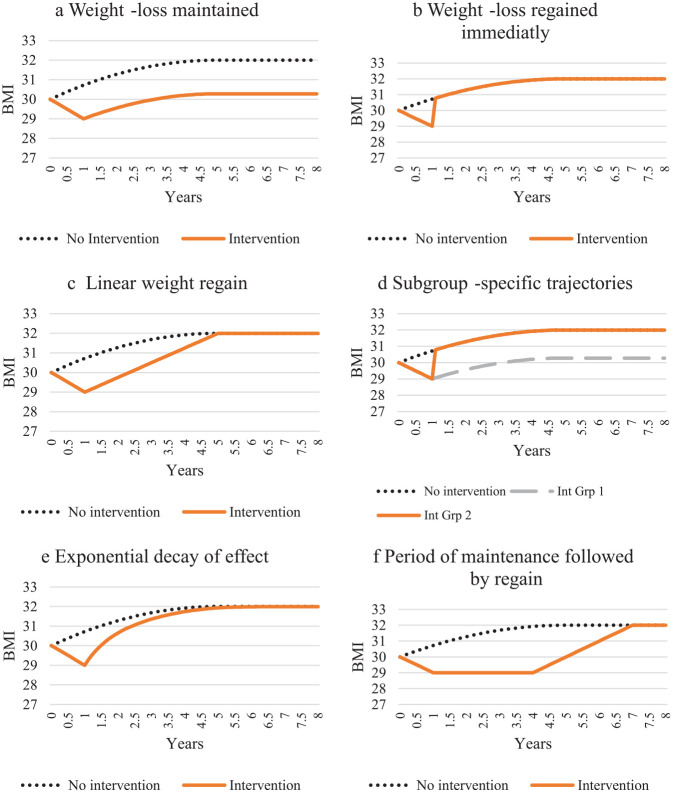
Graphical representations of categories of weight trajectory assumptions used in health economic models of overweight or obesity.

Six different methods were used to predict weight trajectories in the HEMs; these are graphically represented in Figure 2.

#### Weight loss maintained

Twelve HEMs^23–34^ assumed that the weight loss experienced by the intervention group was maintained such that from the end of the trial, and for the remainder of the time horizon, the weight difference between the intervention and control group was maintained. The parallel weight trajectories were either stable (each group remained the postintervention weight) for the remainder of the time horizon^23–28,32,33^ or followed a natural history of weight in which individuals followed the expected trajectory of someone with their postintervention weight^29–31,34^ (Figure 2a).

#### Weight loss regained immediately

Eight HEMs^35–42^ assumed that the intervention effect ceased after the trial follow-up and that those receiving the intervention immediately returned to the same weight as the control group. From this point onward, there was no weight difference between the intervention and control groups; their weight either remained at that value for the remainder of the time horizon^35–39,42^ or followed a natural history trajectory^40,41^ (Figure 2b).

#### Linear weight regain

Eleven HEMs^43–53^ assumed that the weight loss was regained by a set time after completion of the trial or intervention. The time at which all weight was regained varied from 5 months^52^ to 5 years^43^ postintervention (Table 1). Following this, it was assumed that both groups either remained the same weight^43,44,47–49,51,52^ or followed a natural history weight trajectory for the remainder of the time horizon^45,46,50,53^ (Figure 2c).

**Table 1:** Evidence Sources Used to Inform the Prediction of Weight Trajectory

First Author	BMI Trajectory Assumption	Type of Evidence Source	Description and Brief Findings	Limitations
Au^47^	Weight regain between week 26 and 78 in the study was extrapolated until baseline BMI was reached.	Trial^54^	The trial compared 6 months of SBT with detailed meal plans and shopping lists (*n* = 163). One-year postintervention, weight loss was 6.9 kg for the intervention group compared with 3.3 kg for the SBT group.	The sample size was small and had a maximum follow-up of 18 months (12 months postintervention).
Cobiac^55^	Annual exponential decay of effect of 50% (almost no effect after 5.5 years)	Meta-analysis^6^	The review included 46 studies (11,853 participants) examining the impact of dietary counseling interventions on weight loss compared with a control group with follow-up of up to 5 years. Results suggest a regain of 0.02 to 0.03 BMI units per month postintervention such that, on average, participants return to their baseline weight after 5.5 years.	Only a single study (*n* = 51) had a follow-up of 5 years. Studies had high rates of missing data and were moderate to poor quality.
Cleghorn^52^	Weight regain of 0.03 BMI unit/month (regained fully by 5 months postintervention)			
Forster^43^	Weight regain of 0.03 BMI unit/month (regained fully by 5 years postintervention)			
Fuller^48^	Weight regain of 0.03 BMI unit/month after the 2-year follow-up			
Retat^50^	All weight loss was regained over 5 years postintervention.			
Whelan^44^	Weight regain of 0.03 BMI unit/month			
Ginsberg^56^	Annual exponential decay of effect of 50%	Meta-analysis^57^	The review included 80 studies (26,455 participants) of weight loss interventions with at least 1-year follow-up. Approximately 50% of weight loss was regained at 24, 36, and 48 months.	The meta-analysis was conducted on only 21 diet and/or exercise studies (the remainder were pharmacological interventions). The average proportion of participant dropout was 29%.
		Trial^5^ (also referenced by Gillet et al^58^)	The diabetes prevention program (US) examined the effectiveness of an intensive lifestyle intervention for 3234 overweight individuals. Participants lost a mean of 7 kg by 1 year. This was gradually regained, and at the 7-year follow-up, participants maintained at weight loss of 2 kg.	Only individuals with impaired glucose tolerance were included. Lifestyle sessions to reinforce original weight loss were offered every 3 months, which may have increased weight loss maintenance. At the 3-year follow-up, weight was collected from less than 50% of participants.
		Observational study^8^ (also reference by Roux et al.^59^)	The national weight control registry is a large (*n* > 4000) self-selecting sample of individuals who had successfully maintained weight loss (≥13.6 kg) for at least 1 year at entry into the registry. Participants in this study reported having lost an average of 33 kg from an average maximum weight of 105 kg. More than 87% of participants reported maintaining a weight loss of at least 10% (of initial weight) after 10 years.	Participants were self-selecting, and weight loss on entry to the registry and weight change while in the registry were self-reported.
Gillet^58^	Responders (40%) maintained weight loss until year 4 and regained all weight loss by year 8.	Trial^60^	The Finnish Diabetes Prevention Study (*n* = 523) examined the effectiveness of a diabetes prevention lifestyle (diet plus exercise) intervention. At the 7-year follow-up, the intervention group had maintained an average weight loss of 3.1 kg (maximum average weight loss reported at 2 years to be 4.2 kg).	The mean follow-up was 3.2 years, indicating longer follow-up was not available for many participants. Only individuals with impaired glucose tolerance were included.
Galani^61^	Weight loss maintained until year 6 before a linear weight regain to year 10			
Galani^62^	Weight loss maintained until year 6 before a linear weight regain to year 10			
Kent^49^	Weight returned to baseline weight over 5 years	Meta-analysis^7^	The review included 45 trials (7788 participants) of behavioral interventions focused on weight loss maintenance. The mean difference between the intervention and control groups was significant at 24 months but not at 30 months.	Only 2 studies (*n* = 694) reported outcomes at 24 and 30 months. The average participant dropout was 28.4% and 20% for the weight loss and weight loss maintenance interventions, respectively.
Lymer^53^	Participant’s weight increased by 3% annually from their lowest weight to their preintervention weight.	Trial^63^	In a comparison of a 12-month commercial weight-management intervention and standard care (*n* = 772), there was no significant weight difference between groups at 24 months.	Follow-up was limited to 24 months (1-year postintervention). Only 203 of 772 participants completed the 24-month visit.
Roux^59^	Participants had a 20% probability of long-term weight maintenance (remain at postintervention weight for the remainder of the time horizon) and a 67% probability of short-term weight maintenance (weight maintenance for 6 months). The remainder did not lose weight.	Observational study^73^	A telephone survey of participants who had maintained a weight loss of at least 10% from their maximum weight for at least a year. Of those who had been overweight (*n* = 228), 62% reported losing more than 10% of their maximum weight and of these, 47%–49% had maintained the weight loss for at least 1 year.	The sample size was small and all weight change was self-reported. Only 57% of people contacted agreed to take part in the survey.
		Trials	Lowe et al.^64^ examined weight loss maintenance among participants (*n* = 1002) of a commercial weight loss program. At 5 years, 42.6% of participants had maintained a loss of 5% or more, and 18.8% had maintained a loss of 10% or more.	All participants had already met their goal weight (determined by the participant); maintenance among participants who did not meet their goal weight was not included.
			Anderson et al.^65^ assessed long-term weight maintenance after a very-low-calorie dietary intervention. Participants (*n* = 122) regained an average of 73% of their weight loss during the first 3 years. The average weight loss maintained was 23% of initial weight loss after 5 years.	The sample size was small. There were 426 participants in the program, but only 154 were eligible for follow-up (e.g., completed the program and met weight loss target of 10 kg), and data were available for only 122 (73%) of these.
			Gosseline and Cote^66^ reported weight loss maintenance among participants (*n* = 291) of a commercial weight loss program. At a follow-up of 9 to 11 years, 20% maintained at least 5% of their initial weight.	A maximum of 55 participants completed assessments at each time point. Only participants who had reached their goal weight in the initial weight loss program were included.
		Meta-analysis^67^	The review included 29 studies (4298 participants) of dietary interventions. At 5 years postintervention, the average weight maintenance was 23% of initial weight loss.	Only very low-energy or energy-balanced dietary interventions were included. Eight (1388 participants) of the 29 studies had a 5-year follow-up. An average of 79% of participants were available for follow-up.
Segal^68^	Successful participants (33%) maintained weight loss until year 4, when all weight was regained. The remainder followed the trajectory of the control group.	Trial^69^	In a feasibility trial of 370 participants with impaired glucose tolerance, participants (90% available for follow-up) maintained an average of 50% of initial weight loss after 5 years.	The sample size was small and limited to participants with impaired glucose tolerance.

BMI, body mass index; SBT, standard behavioral therapy.

#### Subgroup-specific trajectories

Three HEMs^58,59,68^ divided the population assigned to a weight-management intervention into 2 groups with associated trajectories (e.g., Figure 2d). In one study,^59^ individuals were divided into short-term (6-month) and long-term (5-year) maintainers; the latter were then assumed to maintain this weight for the rest of the time horizon. The probabilities of long- and short-term weight maintenance were 20% and 67%, respectively. Two HEMs^58,68^ divided individuals into responders and nonresponders. Responders were defined as those who successfully lost weight^68^ or successfully maintained the weight loss during the intervention.^58^ The percentage of responders ranged from 33%^68^ to 40%,^58^ and responders were expected to maintain weight loss for 4 years before either regaining the weight immediately^68^ or over a further 4 years to return to preintervention weight by 8 years postintervention.^58^

#### Exponential decay of effect

Two HEMs^55,56^ assumed an annual effect reduction per year (Figure 2e). Ginsberg and Rosenberg^56^ assumed an annual reduction of effect of 50%; in the first year, 50% of the weight loss was regained, and the following year, 50% of the remaining weight loss was regained, and this continued until the effect had effectively diminished. Cobiac and colleagues^55^ did not report the rate at which the intervention effect declined, but they stated that the rate used resulted in almost complete weight regain by 5.5 years after baseline. In both models, it was assumed that the weight of the control group remained stable throughout the time horizon rather than follow a natural history weight trajectory.

#### Period of maintenance followed by regain

Two HEMs^61,62^ assumed that, for those participating in the weight-management intervention, there was a period of weight loss maintenance followed by weight regain (Figure 2f). In both HEMs weight loss was maintained for 6 years and regained between 6 and 10 years and it was assumed that the weight of the control group remained stable throughout the time horizon rather than follow a natural history weight trajectory.

### Evidence Sources Used to Justify Assumed Weight Trajectories

None of the HEMs that included assumptions that either weight loss was maintained (*n* = 12) or regained immediately (*n* = 8) cited an evidence source to justify this assumption. Of those that used other assumptions, 3 did not give an evidence source.^45,46,51^ Of the remaining 15 HEMs, seven^43,44,48–50,52,55^ cited a meta-analysis, six^47,53,58,61,62,68^ cited trials, and two^56,59^ cited a range of sources (including meta-analyses, trials, and observational studies). The details of the evidence sources are provided in Table 1.

Nine of the HEMs^29–31,34,40,41,45,46,50^ used a natural history to represent the weight trajectory of the control group and the intervention group once, and if, weight had been regained. The annual rates of weight gain reported for the natural history trajectories were 1 kg,^30,46^ 0.43 kg,^29^ 0.46 kg,^34^ and 0.16 BMI units^40^; 4 studies did not report this detail.^31,39,45,50^ These rates of regain were based on the change observed in individuals over time within trials,^5,70^ a meta-analysis,^71^ observational studies,^72,73^ or NICE guidelines.^1^

### Impact of Differing Assumptions on Outcomes

Nineteen of the HEMs conducted sensitivity analysis around the assumption of weight trajectories. In these studies, other assumptions about weight trajectories were modeled to determine the magnitude of change in the outcomes. The assumption used in the main analysis and resulting ICER and the sensitivity analysis conducted and corresponding ICER (or reported impact) are reported in Table 2. The findings in this table indicated that the weight trajectory assumption did affect the cost-effectiveness outcomes. In 8 of these studies,^24,26,49,51,52,55,56,59^ the sensitivity analysis had a large enough impact on the outcomes of the evaluation that the ICER crossed a known or estimated cost-effectiveness threshold in the country in which the analysis was based. This may have altered the conclusions and recommendations from the CEA. Five of these tested the scenarios in which all weight loss was either maintained for the remainder of the time horizon^52,55,56^ or regained immediately.^24,26^ Two tested a scenario in which the duration of the intervention effect was reduced,^49,51^ and 1 reduced the probability of individuals achieving weight maintenance.^59^ In another HEM^27^ that tested an increase in the percentage of weight loss regained, the cost-recovery period increased from 6 to 13 years (ICER not reported), which may also affect the assessment of cost-effectiveness.

**Table 2:** Impact of Sensitivity Analyses Conducted on Predicted Weight Trajectories within HEMs

First Author	Method Used to Predict Weight Trajectory	Base-Case ICER	Specific Method Tested in Sensitivity Analysis	Impact on ICER
Au^47^	Linear weight regain	£166/QALY	Upper CI of treatment effect and regain	£61/QALY
			Lower CI of treatment effect and regain	£330/QALY
Bemelmans^36^	Weight regained immediately	€7400/QALY	Permanent decrease in overweight of 1 percentage point and no improvement in physical activity	€9900/QALY
			Permanent decrease of 4% in overweight and inactivity	€5600/QALY
Cleghorn^52^	Linear weight regain	79700 NZD/QALY	Weight loss maintained	Cost saving
Cobiac^55^	Exponential decay of effect	130000 AUD/DALY	Rate of decay varied from no benefit after the first year to full benefit sustained for life	Probability of cost-effectiveness: 0% to 83% (threshold of $50000 /DALY)
Finkelstein^51^	Linear weight regain	$30071/QALY	Duration of intervention effect reduced from 3 years to 1 year	$58867/QALY
Forster^43^	Linear weight regain	12000 AUD/DALY	Rate of regain halved	3000 AUD/DALY
Ginsberg^56^	Exponential decay of effect (annual decay of 50%)	47559 NIS/QALY	Annual decay of intervention effect 20%	11812 NIS/QALY
			Annual decay of intervention effect 35%	29661 NIS/QALY
			Annual decay of intervention effect 65%	65457 NIS/QALY
			Annual decay of intervention effect 80%	83355 NIS/QALY
Gray^34^	Weight loss maintained	£2150/QALY	Weight regained	Remained cost-effective
Gustafson^23^	Weight loss maintained	$183/LYG	50% of weight loss maintained	$3612/LYG
			Weight loss regained after 1 year	$18615/LYG
Hersey^27^	Weight loss maintained	$4400–$5600/QALY (cost-recovery period 6 years)	Participants regained 30% more	Cost-recovery period increased to 13 years
			Participants regained 30% less	No impact on cost-recovery period
Kent^49^	Linear weight regain	£12955/QALY	Participants maintained a 1kg lower weight than their preintervention weight after 5 years	£3203/QALY
			Weight regained immediately and then each year up to 5 years	Cost-effective only if weight regain takes ≥3 years
Krukowski^24^	Weight loss maintained	$2160–$3306/LYG	All participants returned to preintervention weight at 1 year	$73005–$111736/LYG
			Participants regained 50% of the weight at year 1 and the remaining weight by the end of the time horizon	$6602/LYG
Lewis^40^	Linear weight regain	£12585/QALY	Assumed that BMI returned to preintervention weight after 12 months if data were not available	£15276/ QALY
Meads^29^	Weight loss maintained	Dominant	All weight loss regained by year 2	Dominant
			All weight loss regained by year 3	Dominant
Miners^30^	Weight loss maintained	£103112/QALY	Doubled the time to a 0.1 BMI increase after the treatment stops	£122125/QALY
Palmer^38^	Weight regained immediately	£6381/LYG	Intervention effective over lifetime	£4439/LYG
Roux^59^	Subgroup-specific trajectories: probability of short- and long-term maintenance 67% and 20%	$12640/QALY	Probability of long-term maintenance 0%	$36000/QALY
			Probability of long-term maintenance 60%	$5000/QALY
			Probability of short-term maintenance 20%	$130000/QALY
			Probability of short-term maintenance 80%	$15000/QALY
Sacks^26^	Weight loss maintained	Dominant	Effect decayed progressively down to no effect after 10 years	$50000 AUD/DALY
Trueman^46^	Linear weight regain	Dominant	Weight loss is maintained as a decrement below the expected weight trajectory	Dominant

AUD, Australian dollars; BMI, body mass index; CI, confidence interval; DALY, disability-adjusted life-year; HEM, health economic model; ICER, incremental cost-effectiveness ratio; LYG, life-year gained; QALY, quality-adjusted life-year; NIS, Israeli New Shekel; NZD, New Zealand dollars.

**Table 3:** Psychosocial Variables Measured within Evidence Sources Referenced in Health Economic Models

Variable Measured	Definition	Measured in Evidence Source Cited for Estimated:	
		Weight Loss	Weight Regain
Depression	Persistent low mood and loss of interest or pleasure^74^	Ahern,^45^ Forster,^43^ Fuller,^48^ Gustafson^23^	Ginsberg,^56^ Roux^59^
Anxiety	Feelings of tension, worry, or unease with physical symptoms such as sweating^74^		
Dietary restraint	Conscious restriction of dietary intake to manage weight^75^	Ahern,^45^ Forster,^43^ Fuller,^48^ Lymer^53^	Ginsberg^56^
Social support	The quantity and quality of people that an individual feels they can rely on and seek support from^76^	Cecchini,^41^ Forster,^43^ Fuller,^48^ Gustafson^23^	
Dietary disinhibition	The tendency to overeat in response to factors such as availability of palatable foods or emotional stress^75^	Forster,^43^ Fuller,^48^ Lymer^53^	Ginsberg^56^
Binge eating	The extent to which an individual consumes more than most would and feels out of control when eating^77^		Ginsberg,^56^ Roux^59^
Health attitudes	Beliefs, feelings, and thoughts about food (e.g., beliefs about what is healthy or that diet is important for health^78^	Cecchini,^41^ Forster^43^	
Perceived stress	The extent to which situations in an individual’s life are viewed as stressful^79^	Forster^43^	Ahern,^45^ Retat^50^
Habit	The extent to which health behaviors become automatic and part of an individual’s identity^80^	Ahern^45^	Ahern,^45^ Retat^50^
Self-regulation	Monitoring of own health behavior, which can be autonomous (internally motivated) or controlled (externally motivated)^45^	Ahern^45^	
Problem eating behavior	The perception of certain eating behaviors as problematic to the individual^45^	Ahern^45^	Roux^59^
Life satisfaction	The extent to which an individual is satisfied with their life^81^	Ahern^45^	
Self-monitoring	The degree to which an individual records or monitors the food they consume and the exercise they do^82^	Au^47^	
Resources	The financial, cognitive, and time resources that an individual has available to them	Au^47^	
Self-efficacy	An individual’s belief in his or her ability to execute healthy eating and exercise behaviors^83^	Cecchini^41^	
Outcome expectancies	An individual’s belief that a certain behavior or action will lead to a specific outcome^84^	Cecchini^41^	
Hedonic hunger	The drive to eat for pleasure in the absence of a physiological need for food^85^		Ginsberg^56^
Self-esteem	The way an individual positively or negatively evaluates themselves^86^	Gray^34^	Roux^59^
Mood	An individual’s state of mind or feeling^87^		Roux^59^
Affect (positive and negative)	The emotions and expression of a positive (e.g., cheerfulness) or negative (e.g., sadness) nature^88^	Gray^34^	

### Factors Used to Predict Weight Trajectories

None of the studies reported using psychosocial factors to predict weight trajectories.

### Measurement of Psychosocial Factors within Evidence Sources Informing Weight Trajectories

The evidence sources cited for 1) estimated weight loss and 2) estimated weight regain trajectory were examined to determine if any psychosocial variables had been measured. Psychosocial variables measured in either of these indicate the potential to have included these within the health economic modeling to inform predictions of weight trajectories.

#### Estimated weight loss

Thirty HEMs cited an evidence source for estimated weight loss that reported no measurement of psychosocial variables.^24–33,35–40,42,44,46,49–52,55,56,58,59,61,62,68^ Psychosocial variables were measured in evidence sources cited in 8 HEMs (Table 3); 4 of these HEMs^43,45,48,53^ each based the estimated weight loss on a single trial, but no analyses of the psychosocial variables measured in relation to the intervention or weight change were reported in the trial. Four HEMs^23,34,41,47^ cited 5 trials that included some analysis of psychosocial factors. In 4 of these trials, there were significant changes to psychosocial variables but no reported analysis of the association between these changes and weight loss outcomes. First, in a study that provided either a shopping list for healthy meal ingredients or the ingredients free of charge, there was greater adherence to self-monitoring of food intake and exercise,^47,54^ and both interventions reduced the time and effort required to decide on and plan meals. For those that provided food free of charge, potential financial barriers to healthy eating were reduced.^47,54^ Second, a work-based dietary intervention influenced diet-related attitudes including a reduction in confusion about what to eat and an increase in the belief that food is important for health. There was no reported impact of this intervention on perceived social support or self-efficacy for increasing fruit and vegetable consumption.^78^ Third, a behavioral intervention aimed at low-income women improved perceived social support,^89^ and fourth, an intervention for men delivered through professional football clubs improved self-esteem and positive affect (i.e., feelings and emotions).^34^ One trial reported analysis of associations between psychosocial variables and BMI; following the introduction of a nutritional labeling policy, health attitudes, including beliefs about own health and desire to change health status, were not associated with a change in BMI.^90^

#### Estimated weight regain trajectory

When examining the evidence sources used to estimate the weight trajectory beyond the initial weight loss, 2 HEMs^56,59^ cited studies that included psychosocial variables. In these studies, decreases in dietary restraint^91^ and increases in dietary disinhibition^91,92^ hunger,^91^ depression,^91,92^ and binge eating^91,92^ were associated with regaining weight. Two HEMs^45,50^ cited changes in weight over time observed in the Health Survey for England (HSE) to support the use of an annual weight change for both the control group and intervention group postregain; this is the weight trajectory expected in the absence of any intervention. The HSE is an annual repeat cross-sectional survey of about 8000 adults and included measures of stress and eating habits. The measure of eating habits used was a descriptive measure of eating behavior rather than the extent to which a behavior is habitual. Both stress and eating habits have the potential to affect weight loss maintenance,^13,15,73^ but no analyses were reported to test this.

Overall, the most frequently assessed variables were depression and/or anxiety (*n* = 6), dietary restraint (*n* = 5), and social support (*n* = 4). There was evidence to indicate that dietary restraint, dietary disinhibition, hunger, depression, and binge eating were associated with change in BMI, although only 3 of the 13 evidence sources cited included analyses of the association between the psychosocial variables measured and weight loss outcomes.

## Discussion

There was a wide range of weight trajectory assumptions made within the HEMs, which varied in complexity from simple assumptions such as regaining or maintaining all weight loss to more complex assumptions such as subgroup-specific trajectories or applying an exponential decay of intervention effect. In the absence of data, it is difficult to determine which is the most likely to be accurate. Thus, the second aim was to examine the evidence on which these assumptions are based. Fifteen of the 38 studies included in the review cited an evidence source to justify the assumption made, and these sources included meta-analyses, trials, and observational studies. While many of these sources included a large number of participants and long-term follow-up, the sample sizes decreased as the length of follow-up increased. Furthermore, some of the evidence sources were focused mainly or solely on those participants who were successful in weight loss and weight loss maintenance.^8,60,91,92,93^ Although in 2 HEMs these sources were used to inform the trajectories of successful participants only, another included it alongside other evidence sources to inform the trajectories of all participants, which could result in an overestimation of effect. Others focused on a population with impaired glucose tolerance, and these may have a different weight trajectory to those who have a healthy glucose tolerance given the differences in weight loss observed between those with and without diabetes.^6^ In addition, the evidence sources indicated a wide range of results; estimated weight regain at 5 years ranged from 0% to 100% of initial weight loss, and 1 source reported that more than 80% of participants were able to maintain a 10% (of initial weight) weight loss for 10 years.^94^ There was no evidence cited to support the assumptions that all participants regained weight loss immediately postintervention or maintained all weight loss indefinitely, indicating that these assumptions should not be used within HEMs unless there is strong evidence to support this. However, because of the large variation in reported weight loss maintenance, there is not a single weight trajectory assumption that can be recommended at this time. This justifies further analysis of the factors associated with weight loss maintenance to understand this variation and improve the prediction of weight trajectories.

For the third aim, we reviewed any sensitivity analyses conducted around weight trajectory assumptions. Using different weight trajectories affected the costs and consequences to the extent that, in almost half of the studies that conducted this type of sensitivity analysis, it would likely affect assessments of cost-effectiveness. This highlights that a change in the assumptions used could have a large impact on results and that results from models using different assumptions are unlikely to be comparable. Given this impact, sensitivity analysis on the weight trajectory should always be conducted in health economic modeling of obesity, particularly on the time postintervention at which a participant returns to their preintervention weight (if at all). This is especially important if the main assumption is that all weight loss is immediately regained postintervention or maintained for the rest of the time horizon; there is little evidence for these assumptions, and when tested in sensitivity analysis, they often resulted in large changes in outcomes. The impact that these assumptions had on outcomes further supports the need to gain a greater understanding of weight trajectories.

Reviews of survival analyses used in cost-effectiveness analyses have identified similar limitations in long-term extrapolation methods. Similar to weight trajectories, the long-term survival of individuals is hard to determine from short-term data, has a potentially large impact on estimates of cost-effectiveness, and methods used are not consistent and often not justified.^95,96^ Hawkins and Grieve^96^ stated that considering causal assumptions is essential to improving the accuracy of cost-effectiveness analyses; in survival analyses, these may be factors such as time taken for illness to progress to a more severe state, whereas for the assumptions made about weight trajectories, these may be psychosocial factors.

The fourth and fifth aims of this review were to examine the use of psychosocial variables to predict weight trajectories and the potential role of psychosocial factors in HEMs. None of the HEMs used any psychosocial variables in the prediction of individual weight trajectories. However, psychosocial variables were measured within the evidence sources that informed weight trajectories. Furthermore, analyses conducted within these evidence sources indicated that the weight loss interventions were associated with improvement in self-monitoring, financial and time resources, attitudes, and social support and that decreased dietary restraint and increased dietary disinhibition hunger, depression, and binge eating were associated with weight regain. The variables could have been included in the HEMs, which would not only add to the understanding of why an intervention is effective, which can inform future intervention design, but also aid in the prediction of weight trajectories within HEMs. Weight trajectories may be different depending on whether psychosocial factors (that promote weight loss maintenance) have changed during an intervention. For example, in a trial of 2 weight loss programs, despite equivalent outcomes at the end of the 12-week treatment period, the intervention that focused more on habit formation was associated with greater weight loss maintenance after 6 months.^97^ Including psychosocial variables would enable weight trajectory to be based, in part, on the change in psychological variables, and thus, these long-term differences would be represented. Similarly, an individual’s observed shift in psychosocial variables can be used to inform their long-term weight trajectories, which may better reflect the heterogeneity that is observed in the evidence sources cited by the HEMs. Thus, including psychosocial variables has the potential to improve the accuracy of estimates of long-term weight trajectories and therefore the accuracy of cost-effectiveness estimates.

There are some limitations of this review. First, although PRISMA guidelines were followed, we did not measure quality or risk of bias for the studies; the review was focused on a specific aspect of HEMs on which there are no current guidelines; as a result, the review focused on the description of the method rather than the quality. Second, a formal assessment of the evidence used to support assumptions was not conducted, as this was not in the scope of the review. The type of evidence cited and brief details have been included, but future research could apply a formal assessment that would help to determine which assumption is best supported by evidence. Third, although the search was extensive, it focused on academic journals, and thus, there may have been eligible HEMs generated for organizations such as governments, local authorities, or charities that were not included. Also, the criterion that weight loss must be an aim of the intervention may have excluded health economic modelling of prevention programs that measured weight trajectories despite weight loss not being an explicit aim. Similarly, the restriction to English-language journals may have excluded models using alternative methods. Finally, in considering the impact of the different trajectories, the review was limited to the types of sensitivity analysis conducted by the studies. The extent to which the weight trajectory tested in sensitivity analysis diverged from the base-case assumption varied, and alternative comparisons of assumptions may have led to different conclusions.

## Conclusion

The current review has highlighted that 1) there is no consistent assumption made about weight trajectories beyond a weight loss intervention, 2) the evidence of long-term weight maintenance is limited and results are highly variable, and 3) the assumption used has the potential to impact assessments of cost-effectiveness. Furthermore, 4) despite evidence indicating that psychosocial variables are associated with weight loss maintenance, they have not been used to inform the prediction of weight trajectories. This is despite the finding that 5) psychosocial variables have been measured within cited evidence sources. Future research should investigate how psychosocial variables measured within trials and observational studies can be used within HEMs to increase the accuracy of predicted weight trajectories and estimates of cost-effectiveness.

## Supplemental Material

Appendices.rjf_online_supp – Supplemental material for A Systematic Review of Methods to Predict Weight Trajectories in Health Economic Models of Behavioral Weight-Management Programs: The Potential Role of Psychosocial Factors Supplemental material, Appendices.rjf_online_supp for A Systematic Review of Methods to Predict Weight Trajectories in Health Economic Models of Behavioral Weight-Management Programs: The Potential Role of Psychosocial Factors by Sarah Bates, Thomas Bayley, Paul Norman, Penny Breeze and Alan Brennan in Medical Decision Making
