# Supplementary material for: A Systematic Review of Methods to Predict Weight Trajectories in Health Economic Models of Behavioral Weight-Management Programs: The Potential Role of Psychosocial Factors
Source: Med Decis Making. 2019 Dec 2;40(1):90–105. doi: 10.1177/0272989X19889897 (PMC6985993; doi:10.1177/0272989X19889897)
Supplement: Appendices.rjf_online_supp – Supplemental material for A Systematic Review of Methods to Predict Weight Trajectories in Health Economic Models of Behavioral Weight-Management Programs: The Potential Role of Psychosocial Factors [file Appendices.rjf_online_supp.pdf]

APPENDIX 1: FINAL PAPERS

First author	Year	Title
Ahern, A.	2017	Extended and standard duration weight-loss programme referrals for adults in primary care (WRAP): a randomised controlled trial.
Au, N.	2013	The cost-effectiveness of shopping to a predetermined grocery list to reduce overweight and obesity
Avenell, A.	2004	Systematic review of the long-term effects and economic consequences of treatments for obesity and implications for health improvement.
Bemelmans, P.	2008	The costs, effects and cost-effectiveness of counteracting overweight on a population level. A scientific base for policy targets for the Dutch national plan for action.
Cecchini, M.	2010	Tackling of unhealthy diets, physical inactivity, and obesity: health effects and cost-effectiveness.
Cleghorn, C.	2019	Health benefits and cost-effectiveness from promoting smartphone apps for weight loss: Multistate life table modelling.
Cobiac, L.	2010	Cost-effectiveness of Weight Watchers and the Lighten Up to a Healthy Lifestyle program
Dalziel, K.	2007	Time to give nutrition interventions a higher profile: cost-effectiveness of 10 nutrition interventions
Finkelstein, E.	2019	Incremental cost-effectiveness of evidence-based non-surgical weight loss strategies
Forster, M.	2011	Cost-effectiveness of diet and exercise interventions to reduce overweight and obesity
Fuller, N.	2013	Cost effectiveness of primary care referral to a commercial provider for weight loss treatment, relative to standard care: a modelled lifetime analysis
Galani, C.	2007	Modelling the lifetime costs and health effects of lifestyle intervention in the prevention and treatment of obesity in Switzerland.
Galani, C.	2008	Uncertainty in decision-making: value of additional information in the cost-effectiveness of lifestyle intervention in overweight and obese people
Gillet, M	2012	Non-pharmacological interventions to reduce the risk of diabetes in people with impaired glucose regulation: a systematic review and economic evaluation.
Ginsberg, G.	2012	Economic effects of interventions to reduce obesity in Israel

Gray, C.	2018	Long-term weight loss trajectories following participation in a randomised controlled trial of a weight management programme for men delivered through professional football clubs: a longitudinal cohort study and economic evaluation
Gustafson, A.	2009	Cost-effectiveness of a behavioural weight loss intervention for low-income women: The Weight-Wise Program.
Hersey, J. C.	2012	The efficacy and cost-effectiveness of a community weight management intervention: A randomized controlled trial of the health weight management demonstration.
Kent, S.	2019	Is doctor referral to a low-energy total diet replacement program cost-effective for the routine treatment of obesity?
Krukowski, R.	2011	Comparing behavioral weight loss modalities: incremental cost-effectiveness of an internet-based versus an in-person condition
Lewis, L.	2014	The cost-effectiveness of the LighterLife weight management programme as an intervention for obesity in England
Lymer, S.	2018	The population cost-effectiveness of weight watchers with general practitioner referral compared with standard care
Meads, D.	2014	The cost-effectiveness of primary care referral to a UK commercial weight loss programme
Michaud, T.	2017	Cost-effectiveness and return on investment of a scalable community weight loss intervention
Miners, A.	2012	An economic evaluation of adaptive e-learning devices to promote weight loss via dietary change for people with obesity
Olsen, J.	2005	Cost-effectiveness of nutritional counselling for obese patients and patients at risk of ischemic heart disease
Palmer, A.	2000	Applying some UK Prospective Diabetes Study results to Switzerland: the cost-effectiveness of intensive glycaemic control with metformin versus conventional control in overweight patients with type-2 diabetes
Retat, L.	2019	Screening and brief intervention for obesity in primary care: cost-effectiveness analysis in the BWeL trial
Roux, L.	2006	Economic evaluation of weight loss interventions in overweight and obese women
Sacks, G.	2011	'Traffic-light' nutrition labelling and 'junk-food' tax: a modelled comparison of cost-effectiveness for obesity prevention.

Segal, L.	1998	Cost-effectiveness of the primary prevention of non-insulin dependent diabetes mellitus.
Smith, K. J.	2010	Cost-effectiveness analysis of efforts to reduce risk of type 2 diabetes and cardiovascular disease in southwestern Pennsylvania, 2005-2007
Smith, K.J.	2016	Cost effectiveness of an internet-delivered lifestyle intervention in primary care patients with high cardiovascular risk.
Su, W.	2016	Return on Investment for Digital Behavioral Counselling in Patients with Prediabetes and Cardiovascular Disease
Thorpe, K.	2011	Enrolling People with Prediabetes Ages 60-64 In A Proven Weight Loss Program Could Save Medicare $7 Billion Or More.
Trueman, P.	2010	Long-term cost-effectiveness of weight management in primary care.
Whelan, M.	2014	Feasibility, effectiveness, and cost-effectiveness of a telephone-based weight loss program delivered via a hospital outpatient setting
Wilson, K.	2014	Cost-effectiveness of a community-based weight control intervention targeting a low-socioeconomic-status Mexican-origin population

APPENDIX 2: FULL SEARCH STRATEGY

**Search terms (AND, OR, NOT) and truncation (wildcard characters like ⁎)**	*(Obes⁎ OR Overweight) AND (weight loss OR calorie restriction OR diet,reducing OR weight maintenance OR life?style OR healthy lifestyle OR health diet OR health promotion OR weight reduction programs OR weight management OR eating behaviour OR diet OR health diet OR portion size OR serving size OR exercise OR physical activity) AND cost: OR cost benefit analys: OR health care costs) NOT (child)*<br><br>*((((((((obesity[Title/Abstract] OR Overweight[Title/Abstract])) AND ("caloric restriction"[Title/Abstract] OR "calorie restriction"[Title/Abstract] OR diet[Title/Abstract] OR weight maintenance[Title/Abstract] OR weight loss[Title/Abstract] OR weight reduction[Title/Abstract] OR management[Title/Abstract] OR "eating behaviour"[Title/Abstract] OR "eating behavior"[Title/Abstract] OR "health⁎ diet"[Title/Abstract] OR "portion size"[Title/Abstract] OR "serving size"[Title/Abstract] OR exercise[Title/Abstract])) AND (cost[Title/Abstract] OR cost benefit analysis[Title/Abstract] OR health care costs[Title/Abstract] OR economic[Title/Abstract]))) humans*
**Databases searched**	*Medline, Pubmed, Cochrane, NHS EE, Embase, PSYCinfo, Cinahl, Econlit*
**Part of journals searched**	*Title, abstract, keyword, subject heading word.*
**Years of search**	*Up until 24th November2017*
**Language**	*English*

**Types of studies to be included**	*Heath economic evaluation of non-pharmaceutical non-surgica obesity interventions that include some health economic mode*
**Inclusion criteria (why did you include it?**	*Health economic evaluation*<br>*Includes modelling of outcomes beyond available data*<br>*Incudes modelling of at least one behavioural/public health intervention (including disease prevention programmes)*<br>*Weight or BMI is an outcome*<br>*Aimed at adults (18 -65)*
**Exclusion criteria (why did you rule it out?**	*Health economic evaluation of solely drug and pharmaceutical interventions*<br>*Aimed at children or older adults 65+*<br>*Aimed exclusively at population with health condition (e.g. post MI or post-natal)*<br>*If weight or BMI is not measured as an outcome*

APPENDIX 3: DATA EXTRACTION FORM

**Column Headings**
First author
Country
Type of model
Time horizon
Intervention type
Method/assumption used to predict weight trajectory
Sensitivity analysis on trajectory (1 row for each type if more than one)
Impact on CE outcomes
Any mention of psychosocial variables throughout paper (if yes list what variables and what section of paper)
Reference to psychosocial variable in assumptions of weight trajectory (if yes, list the variables and context)
Analysis conducted between intervention and psychosocial variables

Analysis conducted between psychosocial variables and weight trajectory
Evidence sources cited to justify assumptions made about weight trajectories (1 row per evidence source if more than one)
Author and reference (NA if none)
Type of evidence source (e.g. trial, meta-analysis)
Psychosocial variables measured
Analysis conducted between intervention and psychosocial variables
Analysis conducted between psychosocial variables and weight trajectory
